# Increased prevalence of renal cysts in patients with sickle cell disease

**DOI:** 10.1186/s12882-017-0714-3

**Published:** 2017-09-21

**Authors:** Daveena Meeks, Arunraj Navaratnarajah, Emma Drasar, Ounali Jaffer, C. Jason Wilkins, Swee Lay Thein, Claire C. Sharpe

**Affiliations:** 1 King’s College London, Faculty of Life Sciences & Medicine, London, UK; 2 Royal Sussex County Hospital, Brighton, UK; 3 Department of Renal Medicine, King’s College Hospital NHS Foundation Trust, London, UK; 4 Red Cell Centre, Whittington Hospital, London, UK; 5 Department of Radiology, The Royal London Hospital, Barts Health NHS Trust, London, UK; 6 Department of Radiology, King’s College Hospital NHS Foundation Trust, London, UK; 7 King’s College London, Faculty of Life Sciences & Medicine, Molecular Haematology, London, UK; 8 National Heart, Lung and Blood Institute, The National Institutes of Health, Sickle Cell Branch, Bethesda, USA; 9 Department of Renal Sciences, Division of Transplantation Immunology and Mucosal Biology, Faculty of Life Sciences and Medicine, King’s College London, London, SE5 9RJ UK

**Keywords:** Sickle cell, Kidney, Cyst, Anaemia, Renal

## Abstract

**Background:**

Early detection and interventions have enabled patients with sickle cell disease (SCD) to live well into adulthood. Consequently, the chronicity of SCD allows for the insidious manifestation of multisystem complications, including renal damage. Cystic renal lesions are commonly incidentally discovered on ultrasound and computerised tomography (CT) imaging of the abdomen. Most are benign simple cysts, however, difficulties may be encountered if infection, rupture, haemorrhage or cancerous changes develop. We aimed to determine whether patients with SCD have a higher prevalence of simple renal cysts compared to non-SCD individuals.

**Methods:**

Data for a group of 223 patients with SCD who had undergone an ultrasound and/or CT imaging of the abdomen were extracted for comparison with 180 control patients (haemoglobin genotype unknown), matched for age and ethnicity. Scans were evaluated for 198 SCD patients and 180 controls.

**Results:**

Renal cysts were found in 58% of the SCD group and 20% of the controls (OR 5.4 (CI 2.6–11.0), RR 2.8 (CI 1.9–4.2)). Bilateral renal cysts were found in 28% of the SCD participants in comparison with 5% of the control group. In those who had one or more cysts identified, the average number of cysts was 3.76 for the SCD group and 1.94 for the controls. Men with SCD were more likely to develop cysts than women (66% vs 53%), as were men without SCD (22% vs 17%).

**Conclusions:**

Simple renal cysts occur more frequently, are more abundant and develop at a younger age in patients with SCD than ethnically-matched controls. Further study of the mechanism underlying cyst formation may shed light on both sickle cell nephropathy and other cystic renal diseases.

## Background

Sickle cell disease (SCD) is one of the most common inherited blood disorders worldwide [1]. It is caused by the presence of haemoglobin S (HbS) which results from the substitution of glutamic acid by valine in the sixth codon of the beta haemoglobin chain. SCD comprises a number of genotypes, the most common being homozygous inheritance of two HbS mutations (HbSS). Other common sickle genotypes include coinheritance of HbS with another haemoglobin variant, HbC (HbSC), and beta thalassaemia (and HbSβ^+^). Patients with HbSS and HbSβ^0^ thalassaemia have similar clinical severities and are often considered as a group termed sickle cell anaemia (SCA) [2].

Expansion of newborn screening and early implementation of comprehensive care has greatly improved childhood survival in well-resourced countries such that almost all newborns in these countries can now expect to survive to adulthood. Improved general medical management has also helped patients with SCD live considerably longer, allowing for the insidious manifestation of multisystem complications and end-organ damage [3, 4]. The heavily vascularised renal system is particularly vulnerable to sickle-related insults leading to a condition termed sickle cell nephropathy (SCN). Renal involvement affects approximately 60% of patients with sickle cell disease (HbSS) at some point during their life although only 10 to 15% of these patients will develop end stage renal failure [5].

The relatively hypoxic and hyperosmolar environment of the inner medulla promotes polymerization of deoxygenated HbS and subsequent sickling of erythrocytes, resulting in impaired renal medullary blood flow, microinfarcts and papillary necrosis. Further to this, the persistent anaemia and high cardiac output lead to glomerular hypertrophy and hyperfiltration. Over time the fine network of vasa recta is destroyed and the swollen glomeruli become sclerosed and renal impairment sets in [5].

Cystic renal lesions are commonly incidentally discovered on ultrasound and computerised tomography (CT) imaging of the abdomen. Most are benign simple cysts (Bosniak Classification Stage 1), however, difficulties may be encountered if infection, rupture, haemorrhage or cancerous changes develop [6]. King’s College Hospital (KCH) is a teaching hospital in London that caters to the healthcare needs of a multi-ethnic community and is one of the leading Haemoglobinopathy service providers in the UK. The Haematology and Renal teams at KCH run a joint SCD/Renal outpatient clinic to care for SCD patients with kidney dysfunction. The clinical experience encountered in this clinic has led us to hypothesize that SCD is associated with a higher prevalence of simple renal cysts [7].

## Methods

A cross-sectional study of the ultrasound and computerised tomography (CT) abdominal scans performed on patients with SCD over a 5 year period was carried out at KCH. Two hundred twenty three patients aged 16–59 years, with a clinical diagnosis of sickle cell anaemia (HbSS or HBSβ^0^), were identified as having had an ultrasound or CT abdominal scan from the Electronic Patient Records (EPR). We compared the study population against 180 controls, not known to the sickle cell clinic at KCH, who were closely matched for age and self-reported ethnicity (Black or Afro-Caribbean) and who had presented to Accident & Emergency, within the same 5 year time frame, and undergone CT imaging of the abdomen.

Data on the total number of unilateral and bilateral cysts (> 0.5 cm in diameter) within the kidneys were extracted from the original verified scan reports on EPR. Patients with an estimated Glomerular Filtration Rate (eGFR) of less than 60 ml/min were not included in the study as both the presence of SCD and renal cysts are associated with chronic kidney disease. Two independent radiologists subsequently re-reported all CT scans to specifically look for renal cysts (>0.5 cm in diameter), and rendered a consensus opinion if their assessments differed.

Statistical analyses were performed on 198 SCD participants (111 female, 87 male) and 180 controls (53 female, 127 male) and deemed eligible for inclusion in this study. Associations between SCD and renal cysts were analysed by logistic regression using IBM SPSS statistics software. Odds ratios (OR), relative risks (RR) and 95% confidence intervals (CI) were calculated for the prevalence for renal cysts amongst the SCD group compared with the controls. The frequency with which renal cysts were noted on imaging was assessed using chi-squared test comparing 2 methods at a time. This retrospective audit of imaging was not defined as a research study according to the NHS ‘defining research’ decision tool and therefore in the UK there was no requirement for a submission to be made for ethics committee approval.

## Results

Two hundred twenty three homozygous SCD patients were identified as having had an ultrasound or CT abdominal scan. Twenty five patients were ineligible for inclusion in this study, 24 had an eGFR <60 ml/min and one had no eGFR data. One hundred ninety eight SCD patients met the inclusion criteria, of which 43 had undergone CT abdominal scans and 155 had undergone ultrasonography. These data were compared against the CT abdominal scans of 180 controls (Tables 1 and 2).

**Table 1:** Age and gender distribution of the SCD cohort and control patients

Age	SCD	Controls
<20	40 (36% M)	47 (85% M)
20–29	67 (44% M)	46 (76% M)
30–39	52 (27% M)	31 (61% M)
40–49	26 (23% M)	46 (54% M)
50–59	13 (54% M)	10 (80% M)
Total	198 (44% M)	180 (71% M)

*M* Male

**Table 2:** Prevalence of Renal Cysts in sickle cell disease group (SCD) group versus ethnically-matched controls

	SCD (% of cohort)	Control group (% of cohort)	Odds ratio (95% CI)	Relative Risk (95%CI)
>1 renal cyst (no. with bilateral cysts)	58%	20%	5.4 (2.6–11.0)	2.8 (1.9–4.2)
Average number of cysts (in patients with >1 cyst)	3.76	1.94		

*CI* Confidence interval

**Fig. 1:**
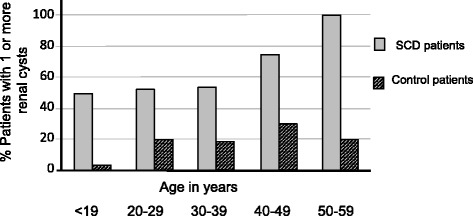
Prevalence of one or more renal cysts in patients with sickle cell disease (SCD) versus controls stratified by age using re-reported CT imaging

**Fig. 2:**
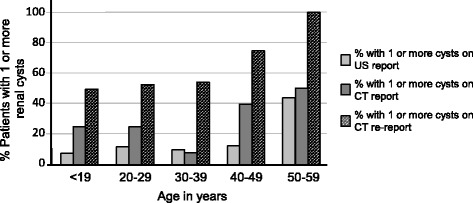
Prevalence of one or more renal cysts in SCD patients stratified by age and imaging method (ultrasound (US) initial report, computer tomography (CT) initial report or CT re-report)

Re-reports of CT scans, confirmed renal cysts in 58% of the SCD group and 20% of the controls (OR 5.4 (CI 2.6–11.0), RR 2.8 (CI 1.9–4.2)). Bilateral renal cysts were found in 28% of the SCD participants in comparison with 5% of the control group. Although men with SCD were more likely to have renal cysts than women (66% vs 53%) this difference was not statistically significant. Similarly, in the control group, 22% of men vs 17% of women had cysts but this was also not significant. On average, the number of cysts identified (in those who had cysts) was 3.76 for the SCD group and 1.94 for the control group. The prevalence of renal cysts increased with age amongst the SCD group, and to a lesser extent in the control group (Fig. 1). Original CT reports mentioned the presence of renal cysts in patients with SCD 2-fold more frequently than did ultrasound reports (*p* < 0.05). Re-reporting of CT scans to look specifically for cysts confirmed their presence in 2.8 fold more patients than had been suggested from the original reports (*p* < 0.05) (Fig. 2).

## Discussion

In this study, confirmed renal cysts were present in more than half of the SCD group (58%) compared to only a fifth of controls. In addition, in those patients who had one or more renal cysts, SCD patients had higher numbers compared to those found in the control group. Previous studies on the prevalence of simple renal cysts on CT imaging have demonstrated that they are more frequently detected with increasing age and that they are more prevalent in men, and our results are in keeping with this [8, 9] (Fig. 1). Carrim et al. studied images from 617 patients, though they were significantly older than our study groups (average age for women 62.8 years and 65.5 for men) with only 49 patients (8%) being below the age of 40 and 216 (35%) below 60. It is worth noting that only 8% of those under the age of 40 had detectable renal cysts and 75% of these were female, though the numbers were small. In the 40–60 age group, 27.5% had renal cysts (60% male) and overall, 23% of those under the age of 60 had renal cysts, 58% of whom were male. The prevalence of renal cysts in the under 60 age group was therefore similar to that found in our control population (23% vs 20%). Although our control group had a greater proportion of men than the SCD group (71% vs 44%), the prevalence of cysts in the SCD overall remained significantly higher across all age groups.

We found that incidental simple renal cysts were reported about two-fold more frequently on the original radiologists’ CT abdominal scans compared with the ultrasonography reports. Re-reporting of the CT scans identified nearly three times the number of cysts initially reported (Fig. 2). Ultrasound scans are significantly less sensitive at detecting small renal cysts and the stored images may not be representative of the kidneys as-a-whole, making re-reporting of ultrasound scans unreliable. However, this highlights the issue that renal cysts are routinely under-recognised and under-reported in the clinical setting.

The physiologically hypoxic, hyperosmolar nature of the medulla prompts sickling of dehydrated haemoglobin and vascular occlusion [5, 10]. The toxic release of free heme within the kidney leads to nitric oxide depletion and regional variation in the partial pressure of oxygen in the kidney [11, 12]. Over time, recurrent vaso-occlusion may cause microthrombosis, vasculopathy, and glomerular and tubular ischaemia, leading to SCN, chronic kidney disease (CKD) and end-stage kidney disease (ESKD) [5, 10, 11, 13–15].

Multiple bilateral renal cysts are characteristic of polycystic kidney disease (PKD), and its pathology has been linked to over-activation of Hypoxia-inducible factor 1-alpha (HIF-1α) in the epithelial layer and Hypoxia-inducible factor 2-alpha (HIF-2α) in the cystic wall [16]. Renal impairment secondary to PKD has also been shown to be accelerated in patients who are heterozygous for the HbS gene (sickle cell trait) [17].

There is increasing empirical data indicating the role of Hypoxia-inducible factors (HIF) in the pathogenesis of renal cysts. Hypoxia-inducible factors-1 and -2 (HIF-1 and HIF-2) are heterodimer regulators of oxygen homeostasis, thought to control glycolysis and erythropoietin, respectively. HIFα- and β-subunits sense oxygen deficits and mediate compensatory genetic transcription. Under low oxygen tension or inflammation (both of which are present in the kidney in SCD) HIF-α is activated and combines with the β-subunit (HIF-β) to upregulate cellular adaptation. In the kidney, HIF-1α is widely expressed, whereas HIF-2α is predominately confined to the interstitial fibroblasts and endothelium. HIF-1α is expressed in the nuclei of the tubular epithelium despite hypoxia; HIF-2α, however, is not expressed here [18]. Hydrolysis of HIF-α is suppressed during transient hypoperfusion. Short-term activation of HIF is a prerequisite to tissue survival during transient hypoperfusion. Paradoxically, chronic stimulation may cause renal parenchymal connective tissue deposition and CKD [18, 19]. Additionally, a propensity for abnormal HIF-1α activation under normal oxygen tension, amplification of HIF-1α expression under low oxygen tension and the presence of renal cysts has also been demonstrated in SCD mouse models [20, 21].

The α-subunit (HIF-α) undergoes proteolysis during normoxia by the Von Hippel-Lindau (VHL) tumour suppressor [18] and patients with Von Hippel-Lindau disease (an autosomal dominant syndrome that occurs secondary to germline mutations in the VHL tumour suppressor gene) develop multiple simple renal cysts at a young age and renal cell carcinoma later in life [18, 22]. Double-knockout mouse models for both VHL/HIF-1α and VHL/HIF-2α however do not develop kidney cysts, indicating the importance of both proteins for cyst formation [23].

The strengths of this study lie in the novel identification of cystic renal lesions in patients with SCD when compared to an age and ethnically matched control group. The limitations arise from analysis of point of care imaging, so not all patients had the gold standard of cross-sectional imaging. The retrospective nature of work meant that we were not able to determine whether the participants were symptomatic or had developed cyst-related complications. We acknowledge that the increased prevalence of renal cysts in patients with SCD does not necessarily imply cause and effect. Although the control group had a greater proportion of men than the SCD group, our data is in keeping with that observed in previous studies demonstrating a small preponderance for men to develop renal cysts over women, which does not appear to be further impacted on by the presence of SCD.

## Conclusion

Further study of the VHL/HIFα pathway may help us to develop new treatments for progressive cystic diseases of the kidney. In addition it may help us to understand their significance in patients with SCD, as extensive use of medical imaging will continue to highlight their increased prevalence in these patients.

## Abbreviations

CI: 95% confidence intervals
CKD: Chronic kidney disease
CT: Computerised tomography (CT)
eGFR: Estimated glomerular filtration rate
EPR: Electronic patient records
ESKD: End-stage kidney disease
HbS: Haemoglobin S
HbSC: Haemoglobin SC disease
HbSβ^+^: Beta thalassaemia
HIF: Hypoxia-inducible factors
HIF-1: Hypoxia-inducible factors-1
HIF-1α: Hypoxia-inducible factor 1-alpha
HIF-2: Hypoxia-inducible factors −2
HIF-2α: Hypoxia-inducible factor 2-alpha
HIFα: Hypoxia-inducible factors alpha subunit and
HIFβ: Hypoxia-inducible factors alpha subunit
KCH: King’s college hospital
OR: Odds ratios
PKD: Polycystic kidney disease
RR: Relative risks
SCA: Sickle cell anaemia
SCD: Sickle cell disease
SCN: Sickle cell nephropathy
VHL: Von hippel-lindau
